# Supplementary material for: The interactome of intact mitochondria by cross-linking mass spectrometry provides evidence for coexisting respiratory supercomplexes
Source: Mol Cell Proteomics. 2017 Dec 8;17(2):216–32. doi: 10.1074/mcp.RA117.000470 (PMC5795388; doi:10.1074/mcp.RA117.000470)
Supplement: Supplemental Data [file supp_RA117.000470_133922_0_supp_23978_fzffwf.zip › spectra_annotation/mito_spectra_annotation/323-1-5-1-15-1.pdf]

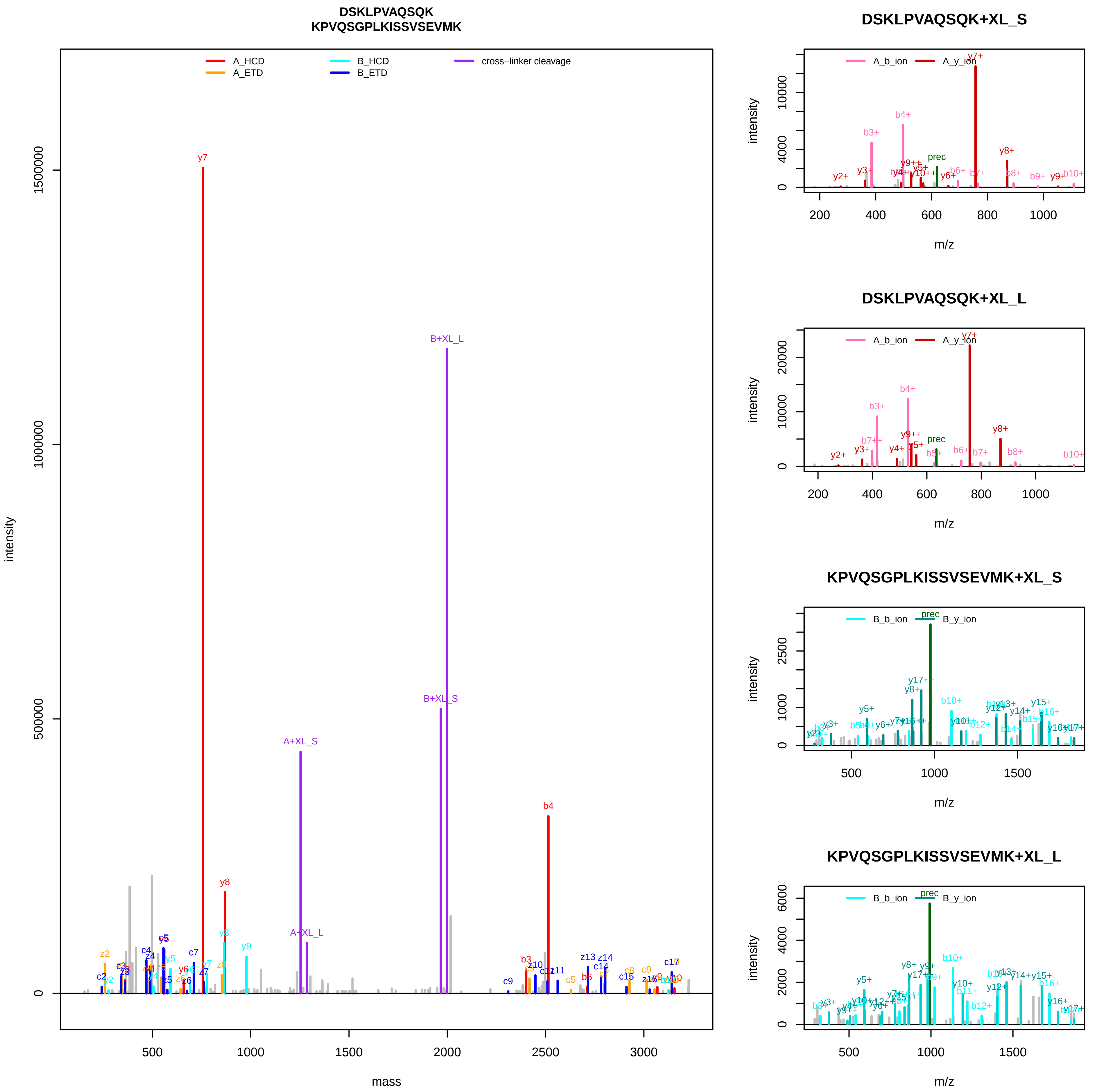
DSKLPVAQSQK
KPVQSGPLKISSVSEVMK
A_HCD
A_ETD
B_HCD
B_ETD
cross−linker cleavage
y7
B+XL_L
B+XL_S
A+XL_S
A+XL_L
y8
b4
y9
intensity
mass
0
500000
1000000
1500000
500
1000
1500
2000
2500
3000
DSKLPVAQSQK+XL_S
A_b_ion
A_y_ion
y7+
b4+
b3+
prec
y8+
intensity
m/z
0
4000
10000
200
400
600
800
1000
DSKLPVAQSQK+XL_L
A_b_ion
A_y_ion
y7+
b4+
b3+
y8+
prec
0
10000
20000
KPVQSGPLKISSVSEVMK+XL_S
B_b_ion
B_y_ion
prec
0
1000
2500
500
1000
1500
KPVQSGPLKISSVSEVMK+XL_L
B_b_ion
B_y_ion
prec
0
2000
4000
6000
